# Maternal and neonatal factors associated with exclusive breastfeeding upon hospital discharge

**DOI:** 10.1590/0034-7167-2025-0196

**Published:** 2025-12-12

**Authors:** Paola Cristina Toapanta-Pinta, Adriana Paola Sinchiguano Cóndor, Deicy Maribel Muso Defaz, Angélica Oliva Gualavisí Landeta, Blanca Llilman Parra Cadena, Santiago Vasco-Morales

**Affiliations:** I Universidad Central del Ecuador, Facultad de Ciencias Médicas. Quito, Pichincha, Ecuador; II Hospital Gineco-Obstétrico Isidro Ayora. Quito, Pichincha, Ecuador

**Keywords:** Breast Feeding, Risk Factors, Infant, Newborn, Maternal Behavior, Maternal and Child Health., Aleitamento Materno, Fatores de Risco, Recém-Nascido, Comportamento Materno, Saúde Materno-Infantil.

## Abstract

**Objective:**

to identify maternal and neonatal factors associated with exclusive breastfeeding at hospital discharge from a tertiary care hospital.

**Methods:**

a cross-sectional study using secondary data from 22,136 records of newborns hospitalized in a neonatal unit (2009-2022). Maternal and neonatal variables and feeding practices at discharge were analyzed. Bayesian logistic regression models were applied.

**Results:**

exclusive breastfeeding at hospital discharge was 91.55%. Negatively associated factors were cesarean delivery (OR: 0.90), illicit substance use (OR: 0.30), low birth weight (OR: 0.84), and cleft lip and palate (OR: 0.15). Breastfeeding counseling and labor and delivery support promoted exclusive breastfeeding at hospital discharge.

**Conclusion:**

despite the high prevalence of exclusive breastfeeding at hospital discharge, barriers persist that must be addressed through promotion strategies, staff training, and support for mothers at risk.

## RESUMO

**Objetivo:**

identificar fatores maternos e neonatais associados ao aleitamento materno exclusivo na alta hospitalar de um hospital terciário.

**Métodos:**

estudo transversal com dados secundários de 22.136 prontuários de recém-nascidos internados na unidade neonatal (2009-2022). Foram analisadas variáveis maternas e neonatais e práticas de alimentação na alta. Modelos de regressão logística bayesiana foram aplicados.

**Resultados:**

o aleitamento materno exclusivo na alta hospitalar foi de 91,55%. Os fatores negativamente associados foram parto cesáreo (OR: 0,90), uso de substâncias ilícitas (OR: 0,30), baixo peso ao nascer (OR: 0,84) e fissura labiopalatina (OR: 0,15). O aconselhamento sobre amamentação e o apoio ao trabalho de parto e parto promoveram o aleitamento materno exclusivo na alta hospitalar.

**Conclusão:**

apesar da alta prevalência de aleitamento materno exclusivo na alta hospitalar, persistem barreiras que devem ser superadas por meio de estratégias de promoção, treinamento da equipe e apoio às mães em risco.

## INTRODUCTION

Exclusive breastfeeding (EBF) is recognized worldwide as the optimal method for ensuring adequate nutrition during the first months of life, promoting healthy growth and development. It also provides maternal health benefits, such as reducing the risk of postpartum hemorrhage and long-term metabolic diseases. In this context, the World Health Organization (WHO) recommends EBF during the first six months of life due to its multiple advantages, such as reducing respiratory and gastrointestinal infections, strengthening cognitive development, and promoting mother-child bonding^(1,2)^.

Despite strong scientific evidence supporting these recommendations, exclusive breastfeeding at hospital discharge (EBHD) rates remain suboptimal in many regions. Factors such as limited access to health services, a lack of effective public policies, and the marketing of infant formulas hinder EBF^(1,3,4)^.

The *Hospital Gineco-Obstétrico Isidro Ayora* (HGOIA) is a national reference institution in Ecuador. For several years, it has implemented the WHO’s Baby-Friendly Hospital Initiative (BFHI)^(5)^ and complies with the national regulations for Mother and Child Friendly Health Establishments (In Spanish, *Establecimientos de Salud Amigos de la Madre y del Niño* - ESAMyN)^(6)^. However, in Ecuador, there is a research gap on the factors that hinder the EBHD of newborns hospitalized in the neonatal area.

## OBJECTIVE

To identify maternal and neonatal factors associated with EBHD in a tertiary care hospital.

## METHODS

### Ethical considerations

The *Universidad Central del Ecuador* Research Ethics Committee approved the study. Informed consent was not required because anonymized secondary data were used.

### Study design and population

A cross-sectional study was conducted based on secondary data from the HGOIA’s Perinatal Information System, a tertiary care hospital and national referral center located in Quito, Ecuador. A total of 22,136 records of mothers and newborns hospitalized in the neonatal unit between January 2009 and December 2022 were analyzed. Variables included maternal and neonatal data as well as feeding practices upon discharge.

This study was reported according to the EQUATOR network guidelines, using the Strengthening the reporting of observational studies in epidemiology guideline^(7)^.

### Inclusion and exclusion criteria

Records of newborns hospitalized in the HGOIA’s neonatal unit and associated information on their mothers were included. Cases with at least one of the following criteria were excluded: newborns transferred to another institution; neonatal death before hospital discharge; mothers with HIV infection; newborns with metabolic diseases that contraindicate breastfeeding (e.g., galactosemia, phenylketonuria); maternal death during hospitalization; incomplete data on key variables (10% missing values for feeding variables at hospital discharge, gestational age, type of delivery, or illicit substance use during pregnancy).

### Variables analyzed

The variable of interest was “diet at hospital discharge”. Maternal variables were analyzed, including sociodemographic characteristics (age, ethnicity, educational level, marital status), obstetric factors (previous pregnancies, pregnancy planning, prenatal visits, breastfeeding counseling, gestational hypertensive disorders, violence during pregnancy, and alcohol, tobacco, and illicit substance use during pregnancy), and childbith aspects (support, delivery route, and general anesthesia). Neonatal variables included sex, gestational age, weight-for-gestational age classification, Apgar scores, multiple pregnancy, and congenital defects (e.g., cleft lip and palate). For statistical analysis, variables were organized into dichotomous or polytomous categories, depending on their nature. Weight-for-gestational age was classified using the Fenton calculator^(8)^.

### Statistical analysis

Absolute and relative frequencies were calculated for sociodemographic, obstetric, neonatal, and delivery variables. A Bayesian approach was used for bivariate and multivariate analyses. This method was selected for its ability to handle unbalanced data and small sample sizes, providing more robust and flexible estimates and minimizing the biases often present in frequentist approaches^(9)^.

The “feeding at hospital discharge” variable was classified into three categories: a) EBF: for newborns fed exclusively with breast milk; b) Partial feeding: for those discharged with breast milk supplemented with formula; c) Artificial feeding: for newborns fed exclusively with formula at discharge. Bayesian analogues were applied to the contingency tables, as well as to the simple and multiple binary logistic regression models, using uninformative priors. For the posterior distribution, 1,000 iterations of the Markov Chain Monte Carlo algorithm were run. The Bayes Factor (BF) was used to assess the evidence between the null and alternative hypotheses (BF > 1 supported the alternative hypothesis; BF < 1 favored the null hypothesis). This probabilistic approach allowed for a more flexible interpretation of the associations between variables and EBF at hospital discharge.

To calculate the Bayesian logistic regression models, the variable of interest was recategorized as dichotomous (EBF (yes/no)). Simple and multiple logistic regression coefficients were estimated, which, when exponentiated, generated unadjusted and adjusted Odds Ratios (ORs) with 95% credibility intervals. The results were interpreted in terms of the posterior probability, which allowed a more precise assessment of the uncertainty associated with the estimates. The analysis was performed using the JASP v0.14.3^(10)^ statistical software, and the trend graph was generated using the R programming language^(11)^.

## RESULTS

During the study period, 26,236 newborns hospitalized in the HGOIA’s neonatal unit were registered. Four thousand one hundred records (15.62%) were excluded for not meeting inclusion criteria: 394 (1.50%) due to transfer to other health institutions; 1,744 (6.64%) due to death before hospital discharge; 118 (0.44%) due to maternal HIV infection; 43 (0.16%) due to maternal death; 11 (0.04%) due to metabolic diseases; and 1,700 (6.47%) due to incomplete data. The final analysis included 22,136 records.

The prevalence of EBHD was 91.55% (N=20,266). Moreover, 6.29% of newborns received partial feeding, while 2.16% received artificial feeding.

**Figure 1:**
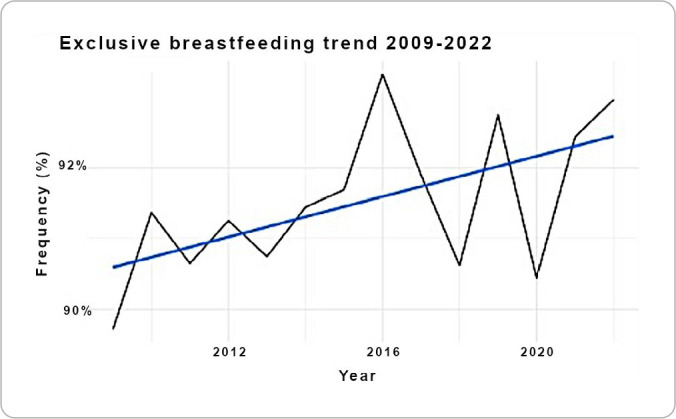
Exclusive breastfeeding trend at the *Hospital Gineco-Obstétrico Isidro Ayora*, Quito, Ecuador (2009-2022)

The trend analysis (Figure 1) shows a progressive increase in the proportion of newborns with EBHD, especially since 2016.

### Characteristics of the study population and comparison of groups with and without exclusive breastfeeding at hospital discharge

Table 1 includes sociodemographic variables, obstetric factors, and birth factors. Most mothers were aged 20 to 29 years, had a secondary education, and were in a stable relationship. A higher frequency of EBHD was observed in mothers with a higher educational level and no history of substance use. Regarding birth characteristics, a higher proportion of EBHD was identified in mothers who delivered vaginally and received breastfeeding counseling during prenatal care.

**Table 1:** Maternal and pregnancy course characteristics of the study population and comparison according to the type of feeding at hospital discharge. *Hospital Gineco-Obstétrico Isidro Ayora*, Quito, Ecuador

Variable	Total population	Comparison of groups			
		Exclusive breastfeeding	Partial	Artificial	BF_10_
	n (%)	n (%)	n (%)	n (%)	
Sociodemographic characteristics					
Maternal age					
Under 20	5,712 (25.80)	5,306 (26.18)	319 (22.92)	87 (18.20)	2.90 × 10^-5^
20 to 29	10,280 (46.44)	9,354 (46.16)	689 (49.50)	237 (49.58)
30 to 39	5,345 (24.15)	4,866 (24.01)	336 (24.14)	143 (29.92)
40 or older	799 (3.61)	740 (3.65)	48 (3.45)	11 (2.30)
**Ethnicity**					
White	158 (0.71)	146 (0.72)	4 (0.29)	8 (1.67)	0.4
Indigenous	458 (2.07)	419 (2.07)	25 (1.80)	14 (2.93)	
Mixed-race	2,0734 (93.67)	19,004 (93.77)	1,306 (93.82)	424 (88.70)	
Black	439 (1.98)	368 (1.82)	46 (3.30)	25 (5.23)	
Other	347 (1.57)	329 (1.62)	11 (0.79)	7 (1.46)	
**Educational level**					
None	303 (1.37)	261 (1.29)	32 (2.30)	10 (2.09)	925.42
Primary	4,944 (22.33)	4,410 (21.76)	381 (27.37)	153 (32.01)	
Secondary	13,396 (60.52)	12,377 (61.07)	765 (54.96)	254 (53.14)	
University	3,493 (15.78)	3,218 (15.88)	214 (15.37)	61 (12.76)	
**Marital status**					
Without a stable partner	5,792 (26.17)	5,363 (26.46)	321 (23.06)	108 (22.59)	0.03
With a stable partner	16,344 (73.83)	14,903 (73.54)	1,071 (76.94)	370 (77.41)	
**Obstetric factors**					
**Previous gestations**					
Yes	13,388 (60.48)	12,160 (60.00)	876 (62.93)	352 (73.64)	99.127.88
No	8,748 (39.52)	8,106 (40.00)	516 (37.07)	126 (26.36)	
**Planned pregnancy**					
Yes	7,110 (32.12%)	6,466 (31.91%)	501 (35.99%)	143 (29.92%)	0.02
No	15,026 (67.88%)	13,800 (68.09%)	891 (64.01%)	335 (70.08%)	
**Prenatal care consultations**					
1 to 4	4,661 (21.06%)	98 (20.50%)	4,212 (20.78%)	351 (25.22%)	3.16x10^13^
5 to 7	9,238 (41.73%)	181 (37.87%)	8,485 (41.87%)	572 (41.09%)	
8 and more	7,323 (33.08%)	153 (32.01%)	6,774 (33.43%)	396 (28.45%)	
None	914 (4.13%)	46 (9.62%)	795 (3.92%)	73 (5.24%)	
**Breastfeeding counseling**					
Yes	10,091 (45.59%)	229 (47.91%)	9,366 (46.22%)	496 (35.63%)	36.14
No	12,045 (54.41%)	249 (52.09%)	10,900 (53.78%)	896 (64.37%)	
**Hypertensive disorders**					
Yes	1,028 (4.64%)	858 (4.23%)	135 (9.70%)	35 (7.32%)	10.19
No	21,108 (95.36%)	19,408 (95.77%)	1,257 (90.30%)	443 (92.68%)	
**Violence during pregnancy**					
Yes	359 (1.62%)	311 (1.53%)	29 (2.08%)	19 (3.97%)	1.33x109
No	21,777 (98.38%)	19,955 (98.47%)	1,363 (97.92%)	459 (96.03%)	
**Alcohol consumption**					
Yes	614 (2.77%)	540 (2.66%)	44 (3.16%)	30 (6.28%)	1.5
No	21,522 (97.23%)	19,726 (97.34%)	1,348 (96.84%)	448 (93.72%)	
**Illicit substance consumption**					
Yes	131 (0.59%)	93 (0.46%)	14 (1.01%)	24 (5.02%)	5.02x10^3^
No	22,005 (99.41%)	20,173 (99.54%)	1,378 (98.99%)	454 (94.98%)	
**Tobacco smoke consumption/exposure**					
Si	1,328 (5.99%)	1,169 (5.77)	111 (7.97)	48 (10.04)	9.99x10^22^
No	20,808 (94.01%)	19,097 (94.23%)	1,281 (92.03%)	430 (89.96%)	
**Factors related to childbirth**					
**Birth route**					
Caesarean section	11,925 (53.87%)	10,847 (53.52%)	704 (50.57%)	374 (78.24%)	9.99E+22
Vaginal	10,211 (46.13%)	9,419 (46.48%)	688 (49.43%)	104 (21.76%)	
**Birth companion**					
Yes	6,834 (30.87%)	6,369 (31.43%)	345 (24.78%)	120 (25.1%)	3.05
No	15,302 (69.13%)	13,897 (68.57%)	1,047 (75.22%)	358 (74.9%)	
**General anesthesia**					
Yes	723 (3.27%)	30 (6.28%)	636 (3.14%)	57 (4.09%)	3.03x10^13^
No	21,413 (96.73%)	448 (93.72%)	19,630 (96.86%)	1,335 (95.91%)	

*Legend: BF_10_: Bayes Factor.*

Table 2 presents the distribution of neonatal characteristics in the study population, categorized according to the type of feeding at hospital discharge. Prematurity, low birth weight for gestational age, and the presence of congenital malformations were more common in infants who received partial or artificial feeding.

**Table 2:** Comparison of neonatal characteristics between exclusively breastfed and non-exclusively breastfed newborns at hospital discharge. *Hospital Gineco-Obstétrico Isidro Ayora*, Quito, Ecuador

Variable	Total population	Comparison of groups			
		Exclusive breastfeeding	Partial	Artificial	BF_10_
	n (%)	n (%)	n (%)	n (%)	
Condition at birth					
Sex					
Female	10,111 (45.68)	9,279 (45.79)	613 (44.04)	219 (45.82)	3.6 × 10^-5^
Male	12,023 (54.31)	10,985 (54.2)	779 (55.96)	259 (54.18)
Not defined	2 (0.01)	2 (0.01)	0 (0.0)	0 (0.0)
**Gestational age**					
Premature	8,043 (36.33)	7,254 (35.79)	606 (43.53)	183 (38.28)	151,25
Break up	13,964 (63.08)	12,900 (63.65)	775 (55.68)	289 (60.46)	
Post-break up	129 (0.58)	112 (0.55)	11 (0.79)	6 (1.26)	
**Weight for gestational age**					
Low	6,717 (30.34)	6,058 (29.89)	471 (33.84)	188 (39.33)	1.873,99
Adequate	14,013 (63.30)	12,918 (63.74)	814 (58.48)	281 (58.79)	
High	1,406 (6.35)	1,290 (6.37)	107 (7.69)	9 (1.88)	
**1-minute Apgar**					
>7	18,576 (83.92)	17,033 (84.05)	1,120 (80.46)	423 (88.49)	3,1
<7	3,560 (16.08)	3,233 (15.95)	272 (19.54)	55 (11.51)	
**5-minute Apgar**					
>7	21,575 (97.47)	19,777 (97.59)	1,331 (95.62)	467 (97.70)	5,34
<7	561 (2.53)	489 (2.41)	61 (4.38)	11 (2.30)	
**Multiple pregnancy product**					
Yes	1,720 (3.89)	1,510 (3.73)	174 (6.25)	36 (3.77)	1.76x10^-5^
No	20,416 (46.11)	18,756 (46.27)	1,218 (43.75)	442 (46.23)	
**Congenital anomalies**					
**Type of defect**					
Major	1,379 (6.23)	1,257 (6.20)	85 (6.11)	37 (7.74)	1.3 × 10^-4^
Minor	938 (4.24)	838 (4.14)	84 (6.03)	16 (3.35)	
None	19,819 (89.53)	18,171 (89.66)	1,223 (87.86)	425 (88.91)	
**Cleft lip and palate**					
Yes	184 (0.83)	116 (0.57)	45 (3.23)	23 (4.81)	4,22x10^23^
No	2,1952 (99.17)	20,150 (99.43)	1,347 (96.77)	455 (95.19)	
**Cardiopulmonary malformations**					
Yes	480 (2.17)	446 (2.20)	23 (1.65)	11 (2.30)	2.39 × 10^-3^
No	21,656 (97.83)	19,820 (97.80)	1,369 (98.35)	467 (97.70)	
**Musculoskeletal malformations**					
Yes	326 (1.47)	302 (1.49)	19 (1.36)	5 (1.05)	1.72 × 10^-3^
No	21,810 (98.53)	19,964 (98.51)	1,373 (98.64)	473 (98.95)	
**Gastrointestinal malformations**					
No	22,029 (99.52)	20,172 (99.54)	1,383 (99.35)	474 (99.16)	0,02
Yes	107 (0.48)	94 (0.46)	9 (0.65)	4 (0.84)	
**Nervous system malformations**					
Yes	615 (2.78)	566 (2.79)	40 (2.87)	9 (1.88)	1.44 × 10^-3^
No	21,521 (97.22)	19,700 (97.21)	1,352 (97.13)	469 (98.12)	
**Urogenital malformations**					
Yes	239 (1.08)	217 (1.07)	19 (1.36)	3 (0.63)	4.6 × 10^-3^
No	21,897 (98.92)	20,049 (98.93)	1,373 (98.64)	475 (99.37)	
**Integumentary malformations**					
Yes	117 (0.53)	108 (0.53)	8 (0.57)	1 (0.21)	5.05 × 10^-3^
No	22,019 (99.47)	20,158 (99.47)	1,384 (99.43)	477 (99.79)	

*Legend: >7: less than; ≥: equal to or greater than; BF_10_: Bayes Factor.*

### Factors associated with exclusive breastfeeding upon hospital discharge

Table 3 shows the results of the logistic regression analysis assessing maternal and neonatal factors associated with BDHD. For this analysis, the “feeding at hospital discharge” variable was recoded as dichotomous (yes/no). Bayesian models estimated odds ratios (crude and adjusted ORs) with their respective 95% Credible Intervals.

**Table 3:** Maternal and neonatal factors associated with exclusive breastfeeding at hospital discharge. *Hospital Gineco-Obstétrico Isidro Ayora*, Quito, Ecuador

Variable	Unadjusted OR	Adjusted OR
**Maternal factors**		
Low educational level	0.68 (0.61 - 0.74)	0.74 (0.66 - 0.82)
Previous pregnancies	0.79 (0.72 - 0.87)	0.97 (0.81 - 1.05)
Alcohol use	0.73 (0.58 - 1.00)	0.94 (0.72 - 1.25)
Illegal substance use	0.23 (0.16 - 0.34)	0.30 (0.20 - 0.46)
Tobacco use/exposure	0.68 (0.57 - 0.80)	0.76 (0.63 - 0.92)
Exposure to violence	0.66 (0.50 - 1.00)	0.83 (0.59 - 1.14)
Prenatal visits ≥5	1.31 (1.17 - 1.43)	1.16 (1.04 - 1.30)
Breastfeeding counseling	1.34 (1.22 - 1.48)	1.28 (1.16 - 1.42)
Hypertensive disorders during pregnancy	0.45 (0.38 - 0.53)	0.39 (0.33 - 0.47)
Partner during labor	1.36 (1.22 - 1.52)	1.38 (1.23 - 1.54)
Cesarean delivery	0.87 (0.78 - 0.95)	0.9 (0.81 - 0.99)
Use of general anesthesia	0.71 (0.59 - 1.00)	0.76 (0.60 - 0.96)
**Neonatal factors**		
Multiple pregnancy	0.65 (0.55 - 0.74)	0.70 (0.60 - 0.84)
1-minute Apgar score <7	0.97 (0.86 - 1.02)	1.01 (0.88 - 1.15)
5-minute Apgar score <7	0.66 (0.51 - 0.87)	0.66 (0.50 - 0.87)
Prematurity	0.77 (0.70 - 0.85)	0.90 (0.82 - 1.01)
Low weight	0.79 (0.73 - 0.88)	0.84 (0.76 - 0.94)
Cleft lip and palate	0.16 (0.12 - 0.21)	0.15 (0.11 - 0.21)

*Legend: <: less than; >: greater than; OR: Odds Ratio.*

## DISCUSSION

The frequency of EBHD in the present study was 91.55%, similar to that reported by Zimmerman *et al*. in the United States, where 91.8% of mothers initiated EBF during their hospital stay^(12)^. However, this proportion differs from that observed in other regions such as Japan, where Yasuda *et al*.^(13)^ documented a prevalence of 66.4%, attributing these results to the lack of support from doulas or midwives. In Turkey, Çelik *et al*.^(14)^ reported 87.6% of newborns discharged with EBF.

Furthermore, 8.45% of newborns did not receive EBHD. Multivariate analysis identified several factors negatively associated with EBHD, such as low maternal educational level, illicit substance or tobacco use during pregnancy, hypertensive disorders, cesarean section, general anesthesia, being a multiple pregnancy, 5-minute Apgar score <7, low birth weight for gestational age, and cleft lip and palate. In contrast, a higher number of prenatal checkups, breastfeeding counseling, and labor and delivery assistance were positively associated with EBHD.

In this study, an increase in EBHD rates was observed, going from 89.71% in 2009 to 93.32% in 2016 and 92.96% in 2022. As a maternal and child hospital, HGOIA follows international regulations, such as the BFHI^(5)^, and national regulations, such as ESAMyN^(6)^, promoting EBF through early initiation, proper use of breast milk substitutes and comprehensive training of health personnel.

Low education showed a negative association with EBHD, coinciding with a study conducted in Cape Verde, where mothers with secondary education or higher were 1.55 times more likely to breastfeed than those with basic education^(15)^.

In this study, illicit substance and tobacco use during pregnancy was negatively associated with EBHD. Local regulations advise against breastfeeding in abstinent mothers while they receive multidisciplinary care, prioritizing their recovery and neonatal well-being^(16)^. Bremer and Knippen reported that opioid exposure before or during pregnancy reduces the likelihood of initiating EBF during hospitalization^(17)^. In the United States, Chang *et al*. identified that 16 of 110 hospitals restrict breastfeeding in mothers with positive cannabinoid results^(18)^.

Beyond hospital policies, substance-using mothers also show lower intentions to breastfeed. Jawale *et al*. observed this trend in women who use marijuana^(19)^. Similarly, smoking during pregnancy and the postpartum period decreases the likelihood of continuing EBF by 51%, according to a study in Sydney, Australia^(20)^. An analysis of the CDC’s Pregnancy Risk Assessment Monitoring System (2016-2018) revealed that combined use of tobacco and illicit substances significantly reduces the likelihood of initiating EBF (aOR: 0.58; 95% CI: 0.39-0.87)^(21)^.

Hypertensive disorders during pregnancy were negatively associated with EBHD. A Canadian study found that these conditions affect the initiation and continuation of breastfeeding^(22)^.

Cesarean section and the use of general anesthesia were negatively associated with EBHD. A cross-sectional study by Pohol *et al*.^(23)^ in Israel found that, in Jewish women of Ethiopian origin, cesarean section reduced EBHD (OR: 0.481; 95% CI: 0.232-0.998). Similarly, Alchalel *et al*.^(24)^ determined that cesarean section reduced the likelihood of EBHD (OR: 0.64; 95% CI: 0.44-0.94; p = 0.023) in hospitals in Haifa and Netanya.

Furthermore, general anesthesia delays skin-to-skin contact, affecting maternal receptivity immediately after surgery. Initiating breastfeeding within the first hour is crucial for the success of EBHD^(25)^.

In this study, being a product of multiple pregnancies was negatively associated with EBHD, likely due to difficulties in timing, organization, and breastfeeding technique. A study in Japan compared EBF among singletons, twins, and triplets, finding a lower prevalence in multiple births. Only 4.1% of twins and triplets received EBF compared to 44.7% of singletons. Furthermore, mothers of twins or triplets were 2.44 times more likely to opt for exclusive formula feeding^(26)^.

A low 5-minute Apgar score was negatively associated with HDB. Herrera Gómez *et al*.^(27)^ found that newborns with a 5-minute Apgar score >7 were 13.4 times more likely to initiate breastfeeding early. This may be due to increased complications and the need for admission to Neonatal Intensive Care Units, which makes it difficult to initiate and maintain HDB.

This study identified a negative association between low birth weight and EBHD. In Ghana, Agyekum *et al*. found that normal birth weight (OR = 7.53; 95% CI: 2.17-26.13) and high birth weight (OR = 6.65; 95% CI: 1.48-29.98) infants were more likely to receive EBF than low birth weight infants^(28)^. Vesel *et al*. identified, in India, Malawi and Tanzania, that maternal perception of insufficient milk supply and latching difficulties were the main barriers to EBF in low birth weight infants^(29)^. In Germany, Scholten *et al*. reported that only 7.8% of mothers initiated breastfeeding immediately, 38.2% within the first six hours, and 60.9% used formula during hospitalization^(30)^.

This study identified a negative association between cleft lip and/or palate and EBHD. Becker de Oliveira *et al*. reported that this condition increases the absence of breastfeeding (OR = 18.08; 95% CI: 7.09-46.09), reduces its frequency (OR = 5.93; 95% CI: 4.30-8.16), and generates greater difficulties in breastfeeding (OR = 13.55; 95% CI: 4.91-37.43)^(31)^. A qualitative study highlighted that hospitalization and the use of tubes, bottles, and syringes hampered the first contact between mother and child, affecting the initiation of breastfeeding^(32)^. Boztepe *et al*. reported that these mothers experienced greater stress due to the inability to breastfeed^(33)^. In Uganda, Nabatanzi *et al*. found that only 72% of children with cleft lip and palate were breastfed, with lack of latch and suction as the main barriers^(34)^. Similarly, Adekunle *et al*. in Nigeria observed that although 83% of mothers initiated breastfeeding, 46% cited inability to suck as the main barrier^(35)^.

Attendance at five or more prenatal checkups and breastfeeding counseling were positively associated with EBHD. Sabilla *et al*. reported that mothers with prenatal care were 1.43 times more likely to achieve EBF compared to those without follow-up (95% CI: 1.416-1.444)^(36)^. Within prenatal care, breastfeeding counseling plays a key role. Lanyo *et al*. found that women who received group prenatal care were more likely to meet the WHO recommendation of EBF for six months (OR: 3.6, 95% CI: 2.1-6.3)^(37)^. Likewise, Lynn & Tener reported that promoting breastfeeding intention was significantly associated with higher breastfeeding self-efficacy (aOR 2.7; 95% CI: 1.3 to 5.8)^(38)^.

In the present study, accompaniment during birth was positively associated with EBHD. García-Bautista^(39)^ found that paternal presence at birth generated positive emotions in women during labor and postpartum, favoring EBF.

No association was identified between previous pregnancies and EBHD. A previous study reported that multiparity is associated with greater use of formula during hospitalization (OR: 1.29; 95% CI: 1.29-1.30)^(40)^, while another found that it favors EBF^(41)^. Similarly, alcohol consumption during pregnancy showed no relationship with EBHD in this study, as did Edwards *et al*., who found no association between prenatal alcohol consumption and EBF^(42)^. No association was identified between violence and EBHD. However, Khalid *et al*. reported that physical and emotional intimate partner violence reduced the likelihood of EBF by 32% (aOR = 0.68; 95% CI: 0.49-0.98; P < 0.05) and 31% (aOR = 0.69; 95% CI: 0.47-0.92; P < 0.05), respectively^(43)^.

Although prematurity was not associated with EBHD in this study, previous research highlights its challenges. Paes *et al*. reported lower breastfeeding rates in late preterm infants compared to full-term infants^(44)^. Crippa *et al*. observed that only 16.8% of late preterm infants in primary care received EBHD^(45)^, while Gianni *et al*. documented a prevalence of 43%^(46)^. Furthermore, Yang *et al*. pointed out that prolonged separation in the Intensive Care Unit makes breastfeeding difficult, being perceived as exhausting despite its importance^(47)^.

### Study strengths

The study has a large sample size and a long analysis period, which strengthens the validity of the findings. Furthermore, its implementation in a national referral hospital provides relevant information for the public health context in Ecuador.

### Study weaknesses

The cross-sectional design prevents establishing causality, limiting it to associations. Retrospective data collection may have compromised data quality. Social desirability bias may have underestimated self-reported variables, such as illicit substance use.

### Contributions to nursing, health, or public policy

The findings guide strategies to improve EBHD rates. Identifying risk factors allows for staff training in complex cases and effective promotion of EBF. Furthermore, they support hospital policies that prioritize monitoring of vulnerable mothers, promote skin-to-skin contact, prenatal education, and breastfeeding-friendly environments. This study contributes to improving maternal and child health indicators and strengthens the evidence base for clinical practice and health policies.

## CONCLUSION

This study highlights the appropriate frequency of EBHD, but also underscores the need to address risk factors that prevent some newborns from benefiting from it. Prospective, preferably multicenter, studies are needed to confirm and investigate new associations that promote or constitute a barrier to EBHD.

In summary, this study provides key evidence to strengthen strategies for promoting, protecting, and fostering EBF.

## Data Availability

The data used in this study are available in Mendeley data: https://data.mendeley.com/datasets/c4wpbkwwx4/1
