# Supplementary material for: Maternal and neonatal factors associated with exclusive breastfeeding upon hospital discharge
Source: Rev Bras Enferm. 2025 Dec 12;78(Suppl 4):e20250196. doi: 10.1590/0034-7167-2025-0196 (PMC12707856; doi:10.1590/0034-7167-2025-0196)
Supplement: Translation

# Translation: Factores maternos y neonatales asociados a la lactancia materna exclusiva al egreso hospitalario

**DOI:** 10.1590/0034-7167-2025-0196es

**Authors:** Paola Cristina Toapanta-Pinta, Adriana Paola Sinchiguano Cóndor, Deicy Maribel Muso Defaz, Angélica Oliva Gualavisí Landeta, Blanca Llilman Parra Cadena, Santiago Vasco-Morales

**Affiliations:** I Universidad Central del Ecuador, Facultad de Ciencias Médicas. Quito, Pichincha, Ecuador; II Hospital Gineco-Obstétrico Isidro Ayora. Quito, Pichincha, Ecuador

**Keywords:** Lactancia Materna, Factores de Riesgo, Recién Nacido, Conducta Materna, Salud Materno-Infantil.

## Abstract

**Objetivo:**

identificar los factores maternos y neonatales asociados con la lactancia materna exclusiva al egreso hospitalario en un hospital de tercer nivel.

**Métodos:**

estudio transversal con datos secundarios de 22.136 registros de recién nacidos hospitalizados en la unidad de neonatología (2009-2022). Se analizaron variables maternas, neonatales y prácticas de alimentación al alta. Se aplicaron modelos de regresión logística bayesiana.

**Resultados:**

la lactancia materna exclusiva al egreso hospitalario fue del 91,55%. Factores asociados negativamente fueron parto por cesárea (OR: 0,90), consumo de sustancias ilícitas (OR: 0,30), bajo peso al nacer (OR: 0,84), y labio y paladar hendido (OR: 0,15). La consejería en lactancia y el acompañamiento en el parto favorecieron la lactancia materna exclusiva al egreso hospitalario.

**Conclusión:**

a pesar de la alta prevalencia de lactancia materna exclusiva al egreso hospitalario, persisten barreras que deben abordarse mediante estrategias de promoción, capacitación del personal y apoyo a madres en situación de riesgo.

## INTRODUCCIÓN

La lactancia materna exclusiva (LME) es reconocida a nivel mundial como el método óptimo para garantizar una nutrición adecuada durante los primeros meses de vida, favoreciendo un crecimiento y desarrollo saludables. Además, proporciona beneficios a la salud materna, como la reducción del riesgo de hemorragia posparto y enfermedades metabólicas a largo plazo. En este contexto, la Organización Mundial de la Salud (OMS) recomienda la LME durante los primeros seis meses de vida, por sus múltiples ventajas, como la disminución de infecciones respiratorias y gastrointestinales, el fortalecimiento del desarrollo cognitivo, y la promoción del vínculo madre-hijo^(1,2)^.

A pesar de la sólida evidencia científica que respalda estas recomendaciones, las tasas de lactancia materna exclusiva al egreso hospitalario (LMEEH) siguen siendo subóptimas en muchas regiones. Factores como el acceso limitado a servicios de salud, la falta de políticas públicas efectivas y la comercialización de fórmulas infantiles obstaculizan la LME^(1,3,4)^.

El Hospital Gineco-Obstétrico Isidro Ayora (HGOIA) es una institución de referencia nacional en Ecuador. Desde hace varios años, ha implementado la Iniciativa Hospital Amigo de la Madre y del Niño (IHAN) de la OMS^(5)^ y cumple la normativa nacional para los Establecimientos de Salud Amigos de la Madre y del Niño (ESAMyN)^(6)^. Sin embargo, en el Ecuador, existe una brecha en la investigación sobre los factores que dificultan la LMEEH de los recién nacidos hospitalizados en el área de neonatología.

## OBJETIVO

Identificar factores maternos y neonatales asociados con la LMEEH en un hospital de tercer nivel.

## MÉTODOS

### Consideraciones éticas

El Comité de Ética en Investigación en Seres Humanos de la Universidad Central del Ecuador aprobó el estudio. Al utilizar datos secundarios anonimizados, no se requirió consentimiento informado.

### Diseño y población del estudio

Se realizó un estudio transversal basado en datos secundarios del Sistema de Información Perinatal del HGOIA, hospital de tercer nivel y referencia nacional ubicado en Quito, Ecuador. Se analizaron 22.136 registros de madres y recién nacidos hospitalizados en neonatología entre enero 2009 y diciembre 2022. Las variables incluyeron datos maternos, neonatales y prácticas de alimentación al alta.

Este estudio fue reportado conforme a las directrices de la red EQUATOR, utilizando la guía *Strengthening the reporting of observational studies in epidemiology* para estudios observacionales en epidemiología^(7)^.

### Criterios de inclusión y exclusión

Se incluyeron los registros de recién nacidos hospitalizados en neonatología del HGOIA y la información asociada de sus respectivas madres. Se excluyeron casos con al menos uno de los siguientes criterios: recién nacidos trasladados a otra institución; fallecimiento neonatal antes del alta hospitalaria; madre con infección por VIH; neonatos con enfermedades metabólicas que contraindican la lactancia materna (ejemplo: galactosemia, fenilcetonuria); muerte materna durante la hospitalización; datos incompletos en variables clave (10% de valores faltantes en las variables de alimentación al alta hospitalaria, edad gestacional, tipo de parto o consumo de sustancias ilícitas durante la gestación).

### Variables analizadas

La variable de interés fue “alimentación al alta hospitalaria”. Se analizaron variables maternas, incluyendo características sociodemográficas (edad, etnia, nivel educativo, estado civil), factores obstétricos (gestas previas, planificación del embarazo, consultas prenatales, consejería sobre lactancia, trastornos hipertensivos gestacionales, violencia durante el embarazo, y consumo de alcohol, tabaco y sustancias ilícitas durante el embarazo) y aspectos del parto (acompañamiento, vía de parto y anestesia general). Las variables neonatales incluyeron sexo, edad gestacional, clasificación del peso para la edad gestacional, puntajes de Apgar, producto de embarazo múltiple y defectos congénitos (ejemplo: labio y paladar hendido). Para el análisis estadístico, las variables fueron organizadas en categorías dicotómicas o politómicas, según su naturaleza. El peso para la edad gestacional se clasificó utilizando la calculadora de Fenton^(8)^.

### Análisis estadístico

Se calcularon las frecuencias absolutas y relativas de las variables sociodemográficas, obstétricas, neonatales y del parto. Para los análisis bivariados y multivariados, se utilizó un enfoque bayesiano. Este método fue seleccionado por su capacidad para manejar datos desbalanceados y tamaños de muestra pequeños, proporcionando estimaciones más robustas y flexibles, y minimizando los sesgos que suelen presentarse en enfoques frecuentista^(9)^.

La variable “alimentación al alta hospitalaria” se clasificó en tres categorías: a) LME: para los recién nacidos alimentados únicamente con leche materna; b) Alimentación parcial: para aquellos que egresaron con lactancia materna complementada con fórmula; c) Alimentación artificial: para los recién nacidos que fueron alimentados exclusivamente con fórmula al alta. Los análogos bayesianos se aplicaron en las tablas de contingencia, así como para los modelos de regresión logística binaria simple y múltiple, empleando priors no informativos. Para la distribución posterior, se ejecutaron 1.000 iteraciones del algoritmo *Markov Chain Monte Carlo*. El Factor de Bayes (BF) se utilizó para evaluar la evidencia entre las hipótesis nula y alternativa (BF > 1 respaldó la hipótesis alternativa; BF < 1 favoreció la hipótesis nula). Este enfoque probabilístico permitió una interpretación más flexible de las asociaciones entre las variables y la LME al alta hospitalaria.

Para el cálculo de los modelos de regresión logística bayesiana, la variable de interés fue recategorizada como dicotómica (lactancia exclusiva (sí/no)). Se estimaron los coeficientes de regresión logística simple y múltiple, los cuales, al exponenciarse, generaron razones de probabilidades (*Odds Ratios*, OR) crudas y ajustadas, con Intervalos de Credibilidad del 95%. Los resultados se interpretaron en términos de la probabilidad posterior, lo que permitió una evaluación más precisa de la incertidumbre asociada a las estimaciones. El análisis se realizó utilizando el *software* estadístico JASP v0.14.3^(10)^, y el gráfico de tendencia fue generado mediante el lenguaje de programación R^(11)^.

## RESULTADOS

Durante el periodo de estudio, se registraron 26.236 recién nacidos hospitalizados en la unidad de neonatología del HGOIA. Se excluyeron 4.100 registros (15,62%) por no cumplir criterios de inclusión: 394 (1,50%) por traslado a otras instituciones de salud; 1.744 (6,64%) por fallecimiento antes del alta hospitalaria; 118 (0,44%) por infección materna por VIH; 43 (0,16%) por muerte materna; 11 (0.04%) por enfermedades metabólicas; y 1.700 (6,47%) por datos incompletos. El análisis final incluyó 22.136 registros.

La prevalencia de LMEEH fue del 91,55% (N=20.266). El 6,29% de los neonatos recibió alimentación parcial, mientras que el 2,16%, recibió alimentación artificial.

El análisis de tendencias (Figura 1) muestra un incremento progresivo en la proporción de recién nacidos con LMEEH, especialmente a partir de 2016.

**Figura 1:** Tendencia de lactancia materna exclusiva en el Hospital Gineco-Obstétrico Isidro Ayora, Quito,Ecuador (2009-2022)

### Características de la población estudiada y comparación de grupos con y sin lactancia materna exclusiva al egreso hospitalario

En la Tabla 1, se incluyeron variables sociodemográficas, factores obstétricos y del parto. La mayoría de las madres tenían entre 20 a 29 años, con educación secundaria y una pareja estable. Se observó una mayor frecuencia de LMEEH en madres con mayor nivel educativo y sin antecedentes de consumo de sustancias. En cuanto a las características del parto, se identificó una mayor proporción de LMEEH en madres que tuvieron parto vaginal y que recibieron consejería sobre lactancia materna en los controles prenatales.

**Tabla 1:** Características maternas y del curso del embarazo de la población estudiada y comparación según el tipo de alimentación al alta hospitalaria. Hospital Gineco-Obstétrico Isidro Ayora, Quito, Ecuador

Variable	Población total	Comparación de grupos			
		Lactancia exclusiva	Parcial	Artificial	BF_10_
	n (%)	n (%)	n (%)	n (%)	
Características sociodemográficas					
Edad materna					
Menor de 20	5.712 (25,80)	5.306 (26,18)	319 (22,92)	87 (18,20)	2.90 × 10^-5^
20 a 29	10.280 (46,44)	9.354 (46,16)	689 (49,50)	237 (49,58)
30 a 39	5.345 (24,15)	4.866 (24,01)	336 (24,14)	143 (29,92)
40 o más	799 (3,61)	740 (3,65)	48 (3,45)	11 (2,30)
**Etnia**					
Blanca	158 (0,71)	146 (0,72)	4 (0,29)	8 (1,67)	0,4
Indígena	458 (2,07)	419 (2,07)	25 (1,80)	14 (2,93)	
Mestiza	2.0734 (93,67)	19.004 (93,77)	1.306 (93,82)	424 (88,70)	
Negra	439 (1,98)	368 (1,82)	46 (3,30)	25 (5,23)	
Otra	347 (1,57)	329 (1,62)	11 (0,79)	7 (1,46)	
**Nivel educativo**					
Ninguno	303 (1,37)	261 (1,29)	32 (2,30)	10 (2,09)	925,42
Primaria	4.944 (22,33)	4.410 (21,76)	381 (27,37)	153 (32,01)	
Secundaria	13.396 (60,52)	12.377 (61,07)	765 (54,96)	254 (53,14)	
Universidad	3.493 (15,78)	3.218 (15,88)	214 (15,37)	61 (12,76)	
**Estado civil**					
Sin pareja estable	5.792 (26,17)	5.363 (26,46)	321 (23,06)	108 (22,59)	0,03
Con pareja estable	16.344 (73,83)	14.903 (73,54)	1071 (76,94)	370 (77,41)	
**Factores obstétricos**					
**Gestas previas**					
Sí	13.388 (60,48)	12.160 (60,00)	876 (62,93)	352 (73,64)	99.127,88
No	8.748 (39,52)	8.106 (40,00)	516 (37,07)	126 (26,36)	
**Embarazo planeado**					
Sí	7.110 (32,12%)	6.466 (31,91%)	501 (35,99%)	143 (29,92%)	0,02
No	15.026 (67,88%)	13.800 (68,09%)	891 (64,01%)	335 (70,08%)	
**Consultas prenatales**					
1 a 4	4.661 (21,06%)	98 (20,50%)	4.212 (20,78%)	351 (25,22%)	3.16x10^13^
5 a 7	9.238 (41,73%)	181 (37,87%)	8.485 (41,87%)	572 (41,09%)	
8 o más	7.323 (33,08%)	153 (32,01%)	6.774 (33,43%)	396 (28,45%)	
Ninguno	914 (4,13%)	46 (9,62%)	795 (3,92%)	73 (5,24%)	
**Consejería sobre lactancia**					
Sí	10.091 (45,59%)	229 (47,91%)	9.366 (46,22%)	496 (35,63%)	36,14
No	12.045 (54,41%)	249 (52,09%)	10.900 (53,78%)	896 (64,37%)	
**Trastornos hipertensivos**					
Sí	1.028 (4,64%)	858 (4,23%)	135 (9,70%)	35 (7,32%)	10,19
No	21.108 (95,36%)	19.408 (95,77%)	1.257 (90,30%)	443 (92,68%)	
**Violencia durante el embarazo**					
Sí	359 (1,62%)	311 (1,53%)	29 (2,08%)	19 (3,97%)	1.33x109
No	21.777 (98,38%)	19.955 (98,47%)	1.363 (97,92%)	459 (96,03%)	
**Consumo de alcohol**					
Sí	614 (2,77%)	540 (2,66%)	44 (3,16%)	30 (6,28%)	1.5
No	21.522 (97,23%)	19.726 (97,34%)	1.348 (96,84%)	448 (93,72%)	
**Consumo de sustancias ilícitas**					
Sí	131 (0,59%)	93 (0,46%)	14 (1,01%)	24 (5,02%)	5.02x10^3^
No	22.005 (99,41%)	20.173 (99,54%)	1.378 (98,99%)	454 (94,98%)	
**Consumo/exposición al humo de tabaco**					
Sí	1.328 (5,99%)	1.169 (5,77)	111 (7,97)	48 (10,04)	9,99x10^22^
No	20.808 (94,01%)	19.097 (94,23%)	1.281 (92,03%)	430 (89,96%)	
**Factores relacionados con el parto**					
**Vía del parto**					
Cesárea	11.925 (53,87%)	10.847 (53,52%)	704 (50,57%)	374 (78,24%)	9,99E+22
Vaginal	10.211 (46,13%)	9.419 (46,48%)	688 (49,43%)	104 (21,76%)	
**Acompañante de parto**					
Sí	6.834 (30,87%)	6.369 (31,43%)	345 (24,78%)	120 (25,1%)	3,05
No	15.302 (69,13%)	13.897 (68,57%)	1.047 (75,22%)	358 (74,9%)	
**Anestesia general**					
Sí	723 (3,27%)	30 (6,28%)	636 (3,14%)	57 (4,09%)	3.03x10^13^
No	21.413 (96,73%)	448 (93,72%)	19.630 (96,86%)	1.335 (95,91%)	

*Leyenda: BF_10_: Factor de Bayes.*

La Tabla 2 presenta la distribución de las características neonatales en la población estudiada, categorizadas según el tipo de alimentación al alta hospitalaria. Se observó que la prematuridad, el bajo peso para la edad gestacional y la presencia de malformaciones congénitas estuvieron más representadas en los neonatos que recibieron alimentación parcial o artificial.

**Tabla 2:** Comparación de características neonatales entre recién nacidos con y sin lactancia materna exclusiva al alta hospitalaria. Hospital Gineco-Obstétrico Isidro Ayora, Quito, Ecuador

Variable	Población total	Comparación de grupos			
		Lactancia exclusiva	Parcial	Artificial	BF_10_
	n (%)	n (%)	n (%)	n (%)	
Condición al nacimiento					
Sexo					
Femenino	10.111 (45,68)	9.279 (45,79)	613 (44,04)	219 (45,82)	3.6 × 10^-5^
Masculino	12.023 (54,31)	10.985 (54,2)	779 (55,96)	259 (54,18)
No definido	2 (0,01)	2 (0,01)	0 (0,0)	0 (0,0)
**Edad gestacional**					
Prematuro	8.043 (36,33)	7.254 (35,79)	606 (43,53)	183 (38,28)	151,25
Término	13.964 (63,08)	12.900 (63,65)	775 (55,68)	289 (60,46)	
Post-término	129 (0,58)	112 (0,55)	11 (0,79)	6 (1,26)	
**Peso para edad gestacional**					
Bajo	6.717 (30,34)	6.058 (29,89)	471 (33,84)	188 (39,33)	1.873,99
Adecuado	14.013 (63,30)	12.918 (63,74)	814 (58,48)	281 (58,79)	
Grande	1.406 (6,35)	1.290 (6,37)	107 (7,69)	9 (1,88)	
**Apgar 1 minuto**					
>7	18.576 (83,92)	17.033 (84,05)	1.120 (80,46)	423 (88,49)	3,1
<7	3.560 (16,08)	3.233 (15,95)	272 (19,54)	55 (11,51)	
**Apgar 5 minutos**					
>7	21.575 (97,47)	19.777 (97,59)	1.331 (95,62)	467 (97,70)	5,34
<7	561 (2,53)	489 (2,41)	61 (4,38)	11 (2,30)	
**Producto de embarazo múltiple**					
Sí	1.720 (3,89)	1.510 (3,73)	174 (6,25)	36 (3,77)	1.76x10^-5^
No	20.416 (46,11)	18.756 (46,27)	1.218 (43,75)	442 (46,23)	
**Anomalías congénitas**					
**Tipo de defecto**					
Mayor	1.379 (6,23)	1.257 (6,20)	85 (6,11)	37 (7,74)	1.3 × 10^-4^
Menor	938 (4,24)	838 (4,14)	84 (6,03)	16 (3,35)	
Ninguno	19.819 (89,53)	18.171 (89,66)	1.223 (87,86)	425 (88,91)	
**Labio y paladar hendido**					
Sí	184 (0,83)	116 (0,57)	45 (3,23)	23 (4,81)	4,22x10^23^
No	2.1952 (99,17)	20.150 (99,43)	1.347 (96,77)	455 (95,19)	
**Malformación cardiopulmonar**					
Sí	480 (2,17)	446 (2,20)	23 (1,65)	11 (2,30)	2.39 × 10^-3^
No	21.656 (97,83)	19.820 (97,80)	1.369 (98,35)	467 (97,70)	
**Malformaciones musculoesqueléticas**					
Sí	326 (1,47)	302 (1,49)	19 (1,36)	5 (1,05)	1.72 × 10^-3^
No	21.810 (98,53)	19.964 (98,51)	1.373 (98,64)	473 (98,95)	
**Malformación gastrointestinal**					
No	22.029 (99,52)	20.172 (99,54)	1.383 (99,35)	474 (99,16)	0,02
Sí	107 (0,48)	94 (0,46)	9 (0,65)	4 (0,84)	
**Malformación de sistema nervioso**					
Sí	615 (2,78)	566 (2,79)	40 (2,87)	9 (1,88)	1.44 × 10^-3^
No	21.521 (97,22)	19.700 (97,21)	1.352 (97,13)	469 (98,12)	
**Malformación urogenital**					
Sí	239 (1,08)	217 (1,07)	19 (1,36)	3 (0,63)	4.6 × 10^-3^
No	21.897 (98,92)	20.049 (98,93)	1.373 (98,64)	475 (99,37)	
**Malformaciones tegumentarias**					
Sí	117 (0,53)	108 (0,53)	8 (0,57)	1 (0,21)	5.05 × 10^-3^
No	22.019 (99,47)	20.158 (99,47)	1.384 (99,43)	477 (99,79)	

*Leyenda: >7: menor que; ≥: igual o mayor que; BF_10_: Factor de Bayes.*

### Factores asociados a lactancia materna exclusiva al egreso hospitalario

La Tabla 3 muestra los resultados del análisis de regresión logística, evaluando factores maternos y neonatales asociados a la LMEEH. Para este análisis, la variable “alimentación al alta hospitalaria” fue recodificada como dicotómica (sí/no). Los modelos bayesianos estimaron las razones de probabilidades (OR crudas y ajustadas) con sus respectivos Intervalos de Credibilidad al 95%.

**Tabla 3:** Factores maternos y neonatales asociados a la lactancia materna exclusiva al alta hospitalaria. Hospital Gineco-Obstétrico Isidro Ayora, Quito, Ecuador

Variable	OR crudo	OR ajustado
**Factores maternos**		
Nivel educativo bajo	0,68 (0,61 - 0,74)	0,74 (0,66 - 0,82)
Con gestas previas	0,79 (0,72 - 0,87)	0,97 (0,81 - 1,05)
Consumo de alcohol	0,73 (0,58 - 1,00)	0,94 (0,72 - 1,25)
Consumo de sustancias ilícitas	0,23 (0,16 - 0,34)	0,30 (0,20 - 0,46)
Consumo/exposición a tabaco	0,68 (0,57 - 0,80)	0,76 (0,63 - 0,92)
Exposición a violencia	0,66 (0,50 - 1,00)	0,83 (0,59 - 1,14)
Consultas prenatales ≥5	1,31 (1,17 - 1,43)	1,16 (1,04 - 1,30)
Con consejería lactancia	1,34 (1,22 - 1,48)	1,28 (1,16 - 1,42)
Con trastornos hipertensivos durante la gestación	0,45 (0,38 - 0,53)	0,39 (0,33 - 0,47)
Con acompañante durante el parto	1,36 (1,22 - 1,52)	1,38 (1,23 - 1,54)
Parto por cesárea	0,87 (0,78 - 0,95)	0,9 (0,81 - 0,99)
Uso de anestesia general	0,71 (0,59 - 1,00)	0,76 (0,60 - 0,96)
**Factores neonatales**		
Producto de embarazo múltiple	0,65 (0,55 - 0,74)	0,70 (0,60 - 0,84)
Apgar <7 al primer minuto	0,97 (0,86 - 1,02)	1,01 (0,88 - 1,15)
Apgar <7 al quinto minuto	0,66 (0,51 - 0,87)	0,66 (0,50 - 0,87)
Prematurez	0,77 (0,70 - 0,85)	0,90 (0,82 - 1,01)
Peso bajo	0,79 (0,73 - 0,88)	0,84 (0,76 - 0,94)
Labio y paladar hendido	0,16 (0,12 - 0,21)	0,15 (0,11 - 0,21)

*Leyenda: <: menor que; >: mayor que; OR: Odds Ratio.*

## DISCUSIÓN

La frecuencia de LMEEH en el presente estudio fue del 91,55%, similar a lo reportado por Zimmerman *et al*. en Estados Unidos, donde el 91,8% de las madres iniciaron la LME durante la estancia hospitalaria^(12)^. Sin embargo, esta proporción difiere de la observada en otras regiones como Japón, donde Yasuda *et al*.^(13)^ documentaron una prevalencia del 66,4%, atribuyendo estos resultados a la ausencia de apoyo por parte de doulas o parteras. En Turquía, Çelik *et al*.^(14)^ informaron de un 87,6% de neonatos dados de alta con LME.

El 8,45% de los neonatos no recibió LMEEH. El análisis multivariado identificó varios factores asociados negativamente con la LMEEH, como el bajo nivel educativo materno, el consumo de sustancias ilícitas o tabaco en la gestación, trastornos hipertensivos, cesárea, anestesia general, ser producto de embarazo múltiple, Apgar <7 al minuto cinco, el bajo peso para la edad gestacional, y labio y paladar hendido. En contraste, un mayor número de controles prenatales, consejería en lactancia y el acompañamiento en el parto se asociaron positivamente con la LMEEH.

En este estudio, se observó un aumento en las tasas de LMEEH, pasando del 89,71%, en 2009, al 93,32%, en 2016, y al 92,96%, en 2022. Como hospital materno-infantil, el HGOIA sigue normativas internacionales, como la IHAN^(5)^, y nacionales, como el ESAMyN^(6)^, promoviendo la LME a través del inicio temprano, el uso adecuado de sustitutos de leche materna y la capacitación integral del personal de salud.

La baja escolaridad mostró una asociación negativa con LMEEH, coincidiendo con un estudio realizado en Cabo Verde, donde las madres con educación secundaria o superior tenían 1,55 veces más probabilidades de amamantar que aquellas con educación básica^(15)^.

En este estudio, el consumo de sustancias ilícitas y de tabaco durante el embarazo se asoció negativamente con la LMEEH. Las normativas locales desaconsejan la lactancia materna en madres en abstinencia mientras reciben atención multidisciplinaria, priorizando su recuperación y el bienestar neonatal^(16)^. Bremer y Knippen reportaron que la exposición a opioides antes o durante la gestación reduce la probabilidad de iniciar LME en la hospitalización^(17)^. En Estados Unidos, Chang *et al*. identificaron que 16 de 110 hospitales restringen la lactancia en madres con resultados positivos de cannabinoides^(18)^.

Mas allá de las políticas hospitalarias, las madres consumidoras de sustancias también muestran menor intención de amamantar. Jawale *et al*. observaron esta tendencia en mujeres que consumen marihuana^(19)^. Asimismo, fumar en la gestación y el posparto disminuye en 51% la probabilidad de continuar la LME, según un estudio en Sídney, Australia^(20)^. Un análisis del Sistema de Monitoreo de Evaluación y Riesgos en el Embarazo de los CDC (2016-2018) reveló que el uso combinado de tabaco y sustancia ilícitas reduce significativamente la probabilidad de iniciar la LME (ORa: 0,58; IC 95: 0,39-0,87)^(21)^.

Los trastornos hipertensivos en el embarazo mostraron asociación negativa con la LMEEH. Un estudio realizado en Canadá encontró que estas condiciones afectan el inicio y la continuidad de la lactancia materna^(22)^.

La cesárea y el uso de anestesia general se asociaron negativamente con la LMEEH. Un estudio transversal realizado por Pohol *et al*.^(23)^ en Israel encontró que, en mujeres judías de origen etíope, la cesárea redujo la LMEEH (OR: 0,481; IC 95: 0,232-0,998). De manera similar, Alchalel *et al*.^(24)^ determinaron que la cesárea reduce la probabilidad de LME (OR: 0,64; IC 95%: 0,44-0,94; p = 0,023) en hospitales de Haifa y Netanya.

Además, la anestesia general retrasa el contacto piel con piel, afectando la receptividad materna inmediata tras la cirugía. El inicio de la lactancia en la primera hora es crucial para el éxito de la LMEEH^(25)^.

En este estudio, ser producto de embarazo múltiple mostró asociación negativa con la LMEEH, probablemente debido a dificultades en tiempo, organización y técnica de amamantamiento. Un estudio en Japón comparó la LME entre neonatos únicos, gemelos y trillizos, encontrando menor prevalencia en nacimientos múltiples. Solo el 4,1% de gemelos y trillizos recibió LME frente al 44,7% de los nacimientos únicos. Además, las madres de gemelos o trillizos tenían 2.44 veces más probabilidades de optar por alimentación exclusiva con fórmula^(26)^.

La baja puntuación de Apgar a los cinco minutos presentó asociación negativa con la LMEEH. Herrera Gómez *et al*.^(27)^ encontraron que neonatos con Apgar >7 a los cinco minutos presentaban 13.4 veces más probabilidades de iniciar la lactancia materna precoz. Esto puede deberse a mayores complicaciones y necesidad de ingreso a Unidades de Cuidados Intensivos Neonatales, lo que dificulta el inicio y mantenimiento de la LMEEH.

Este estudio identificó una asociación negativa entre el bajo peso al nacer y la LMEEH. En Ghana, Agyekum *et al*. encontraron que neonatos con peso normal (OR = 7.53; IC 95: 2.17-26.13) y elevado (OR = 6.65; IC 95: 1.48-29.98) tenían una mayor probabilidad de recibir LME que aquellos de bajo peso^(28)^. Vesel *et al*. identificaron, en India, Malawi y Tanzania, que la percepción materna de insuficiencia de leche y dificultades en el agarre eran las principales barreras para la LME en neonatos de bajo peso^(29)^. En Alemania, Scholten *et al*. reportaron que solo el 7,8% de las madres inició la lactancia de inmediato, el 38,2%, en las primeras seis horas, y el 60,9% utilizó fórmula durante la hospitalización^(30)^.

Este estudio identificó una asociación negativa entre labio y/o paladar hendido y la LMEEH. Becker de Oliveira *et al*. reportaron que esta condición incrementa la ausencia de lactancia materna (OR = 18,08; IC 95: 7,09-46,09), reduce su frecuencia (OR = 5,93; IC 95: 4,30-8,16), y genera mayores dificultades para amamantar (OR = 13,55; IC 95: 4,91-37,43)^(31)^. Un estudio cualitativo destacó que la hospitalización y el uso de sondas, biberones y jeringas dificultaron el primer contacto entre madre e hijo, afectando el inicio de la lactancia materna^(32)^. Boztepe *et al*. informaron que estas madres experimentaron mayor estrés por la imposibilidad de amamantar^(33)^. En Uganda, Nabatanzi *et al*. encontraron que solo el 72% de los niños con fisura labiopalatina fueron amamantados, con la falta de agarre y succión como principales barreras^(34)^. De manera similar, Adekunle *et al*., en Nigeria, observaron que, aunque el 83% de las madres inició la lactancia, el 46% mencionó la incapacidad de succión como el principal obstáculo^(35)^.

La asistencia a cinco o más controles prenatales y la consejería sobre lactancia materna se asociaron positivamente con la LMEEH. Sabilla *et al*., reportaron que las madres con atención prenatal tenían 1,43 veces más probabilidades de lograr LME frente a aquellas sin seguimiento (IC del 95 %: 1,416-1,444)^(36)^. Dentro del control prenatal, la consejería en lactancia desempeña un papel clave. Lanyo *et al*. encontraron que las mujeres con atención prenatal grupal tenían mayor probabilidad de cumplir la recomendación de la OMS de amamantar exclusivamente por seis meses (OR: 3,6, IC del 95%: 2,1-6,3)^(37)^. Asimismo, Lynn & Tener (2024) reportaron que promover la intención de amamantar se asoció significativamente con una mayor autoeficacia en la lactancia materna (ORa 2,7; IC del 95 %: 1,3 a 5,8)^(38)^.

En el presente estudio, el acompañamiento durante el parto se asoció de manera positiva con la LMEEH. García-Bautista^(39)^ encontró que la presencia paterna en el nacimiento generó emociones positivas en las mujeres durante el parto y postparto, favoreciendo la LME.

No se identificó asociación entre gestas previas y LMEEH. Un estudio previo reportó que la multiparidad se asocia con mayor uso de fórmula durante la hospitalización (OR: 1,29; IC 95%: 1,29-1,30)^(40)^, mientras que otro encontró que favorece la LME^(41)^. De manera similar, el consumo de alcohol durante el embarazo no mostró relación con la LMEEH en este estudio, al igual que Edwards *et al*., quienes no encontraron asociación entre el consumo prenatal de alcohol y la LME^(42)^. Tampoco se identificó asociación entre violencia y LMEEH. Sin embargo, Khalid *et al*. reportaron que la violencia física y emocional de pareja redujo la probabilidad de LME en un 32% (ORa = 0,68; IC 95%: 0,49-0,98; P < 0,05) y 31% (ORa = 0,69; IC 95%: 0,47-0,92; P < 0,05), respectivamente^(43)^.

Aunque la prematurez no se asoció con la LMEEH en este estudio, investigaciones previas evidencian sus dificultades. Paes *et al*. reportaron menores tasas de lactancia en prematuros tardíos frente a recién nacidos a término^(44)^. Crippa *et al*. observaron que solo el 16,8% de los prematuros tardíos en el primer nivel de atención recibían LMEEH^(45)^, mientras que Gianni *et al*. documentaron una prevalencia del 43%^(46)^. Además, Yang *et al*., señalaron que la separación prolongada en la Unidad de Cuidados Intensivos dificulta la lactancia, percibiéndose como agotadora pese a su importancia^(47)^.

### Fortalezas del estudio

El estudio cuenta con una muestra amplia y un período de análisis extenso, lo que fortalece la validez de los hallazgos. Además, su realización en un hospital de referencia nacional proporciona información relevante para el contexto de salud pública en Ecuador.

### Debilidades del estudio

El diseño transversal impide establecer causalidad, limitándose a asociaciones. La recolección retrospectiva de los datos pudo afectar la calidad de estos. El sesgo de deseabilidad social pudo subestimar las variables autoinformadas, como el consumo de sustancias ilícitas.

### Contribuciones a la enfermería, la salud o las políticas públicas

Los hallazgos orientan estrategias para mejorar las tasas de LMEEH. La identificación de factores de riesgo permite capacitar al personal en casos complejos y promover eficazmente la LME. Además, respaldan políticas hospitalarias que prioricen el seguimiento de madres vulnerables, fomenten el contacto piel a piel, la educación prenatal y entornos favorables para la lactancia. Este estudio contribuye a mejorar los indicadores de salud materno-infantil, y refuerza la evidencia en la práctica clínica y las políticas sanitarias.

## CONCLUSIÓN

Este estudio destaca que la frecuencia de LMEEH es adecuada, pero también subraya la necesidad de abordar los factores de riesgo que impiden que algunos neonatos se beneficien de ella. Se deben realizar estudios prospectivos y de preferencia multicéntricos para confirmar y buscar nuevas asociaciones que favorezcan o constituyan una barrera para LMEEH.

En síntesis, este estudio proporciona evidencia clave para fortalecer las estrategias de promoción, protección y fomento de la LME.

## Data Availability

Los datos utilizados en este estudio están disponibles en Mendeley data: https://data.mendeley.com/datasets/c4wpbkwwx4/1
